# Biological and Transcriptomic Characterization of Pre-Haustorial Resistance to Sunflower Broomrape (*Orobanche cumana* W.) in Sunflowers (*Helianthus annuus*)

**DOI:** 10.3390/plants10091810

**Published:** 2021-08-30

**Authors:** Dana Sisou, Yaakov Tadmor, Dina Plakhine, Hammam Ziadna, Sariel Hübner, Hanan Eizenberg

**Affiliations:** 1 Department of Phytopathology and Weed Research, Agricultural Research Organization, Newe Ya’ar Research Center, Ramat Yishay 30095, Israel; dinap@volcani.agri.gov.il (D.P.); hammam@volcani.agri.gov.il (H.Z.); eizenber@volcani.agri.gov.il (H.E.); 2 Department of Vegetable and Field Crops, Agricultural Research Organization, Newe Ya’ar Research Center, Ramat Yishay 30095, Israel; tadmory@volcani.agri.gov.il; 3 The Robert H. Smith Institute of Plant Sciences and Genetics, The Robert H. Smith Faculty of Agriculture, Food and Environment, The Hebrew University of Jerusalem, Rehovot 7610001, Israel; 4 Galilee Research Institute (MIGAL), Tel-Hai Academic College, Upper Galilee 11016, Israel; sarielh@migal.org.il

**Keywords:** sunflower (*Helianthus annuus*), broomrape (*Orobanche cumana*), broomrape resistance, transcriptomics, parasitic plants

## Abstract

Infestations with sunflower broomrape (*Orobanche cumana* Wallr.), an obligatory root parasite, constitute a major limitation to sunflower production in many regions around the world. Breeding for resistance is the most effective approach to reduce sunflower broomrape infestation, yet resistance mechanisms are often broken by new races of the pathogen. Elucidating the mechanisms controlling resistance to broomrape at the molecular level is, thus, a desirable way to obtain long-lasting resistance. In this study, we investigated broomrape resistance in a confectionery sunflower cultivar with a robust and long-lasting resistance to sunflower broomrape. Visual screening and histological examination of sunflower roots revealed that penetration of the broomrape haustorium into the sunflower roots was blocked at the cortex, indicating a pre-haustorial mechanism of resistance. A comparative RNA sequencing between broomrape-resistant and -susceptible accessions allowed the identification of genes that were significantly differentially expressed upon broomrape infestation. Among these genes were β-1,3-endoglucanase, β-glucanase, and ethylene-responsive transcription factor 4 (ERF4). These genes were previously reported to be pathogenesis-related in other plant species. This transcriptomic investigation, together with the histological examinations, led us to conclude that the resistance mechanism involves the identification of the broomrape and the consequent formation of a physical barrier that prevents the establishment of the broomrape into the sunflower roots.

## 1. Introduction

Among the plethora of plant pathogens, parasitic weeds are considered a major threat to crops worldwide. Broomrape species (*Orobanche* and *Phelipanche* spp., Orobanchaceae) are obligatory parasitic plants that are particularly damaging to agricultural crops, especially legumes, tobacco, carrot, tomato, and sunflower (*Helianthus annuus* L.). Sunflower broomrape (*Orobanche cumana* Wallr.) thus constitutes a major constraint on sunflower production in many regions around the globe, including the Middle East, Southeast Europe, Southwest Asia, Spain, and China [1]. Because broomrape is a chlorophyll-lacking holoparasite, it obtains all its nutritional requirements from the host plant. The parasitism occurs at the host roots, damaging host development and resulting in significant yield reduction [2]. Broomrape control is a challenging problem because only a few herbicides are effective against broomrape and, more importantly, because the parasite’s attachment to the host root tissues allows systemic herbicides to move from the parasite into the host [2,3]. Therefore, breeding for resistant varieties is the most efficient and sustainable means to control broomrape in sunflower. Generally, there are three types of host resistance to broomrape, in accordance with the developmental stage of the parasitism: The first, a pre-attachment resistance mechanism, depends on the ability of the host to prevent the attachment of the parasite, including the prevention of parasite germination and development, as well as low production or release of germination stimulants [4] such as strigolactones from the host roots into the rhizosphere [5,6]. If pre-attachment resistance fails, broomrape seeds will germinate, and the parasites will grow toward the host roots via chemotropism and attach to the roots [7]. The second resistance mechanism—known as post-attachment or pre-haustorial resistance [4]—is a mechanism inhibiting penetration into the host root cells as well as the development of the haustorium, thus preventing vascular conductivity between the parasite and the host [8]. This resistance involves the production of physical barriers (such as thickening of host root cell walls by lignification and callose deposition) [4,9,10], which prevents the parasite from establishing a vascular connection with the host roots. The third, post-haustorial type of resistance involves the release of a gum-like substance [11,12] and the production and delivery of toxic compounds (phenolics) by the host. The transfer of these chemical compounds to the parasite prevents or delays the formation of the tubercles that are necessary for stalk elongation and flowering of the parasite [11,13,14]. To shed light on the basis of the resistance mechanisms in sunflowers, it is first necessary to understand the structure of the plant innate immunity system. The first level of the plant immune system is pathogen-triggered immunity (PTI), which is activated by the recognition of pathogen-associated molecular patterns (PAMPs). While, over time, pathogens have developed effectors to inhibit the PAMP-activated PTI response, plants, in turn, have evolved to perceive and counteract these effectors through a second layer of defense, known as effector-triggered immunity (ETI), formerly known as gene-for-gene resistance [15]. The rapid changes in the race composition of sunflower broomrape have led to an ongoing gene-for-gene ‘arms race’ between breeders and the parasitic weed. The development of *O. cumana*-resistant cultivars usually includes the introgression of resistance genes, which are, in many cases, broken by the parasite. This resistance breakdown occurs due to the massive use of vertical (monogenic) resistance [16] and can be addressed by the introduction of horizontal (quantitative) resistance genes with the aim of developing a more durable resistance [17,18]. Several *Orobanche* resistance (Or) QTLs that confer resistance to *O. cumana* have been used in breeding programs over the years [19]. These QTLs were numbered (Or1-Or6) in accordance with the gene-for-gene model [20], and they provide resistance against the corresponding *O. cumana* races A to F [19,21,22,23,24]. QTL Or5 was the first to be mapped and was located on the telomeric region of chromosome 3 [25,26,27]. Following this successful attempt to map Or5, a number of studies used the genetic mapping approach to locate more resistance QTLs in sunflower. For example, Perez-Vich et al. (2004) [17] detected eight QTLs for resistance along seven different chromosomes. More recently, Louarn et al. (2016) [28] studied resistance to races F and G and identified a total of 17 QTLs in accordance with different stages of broomrape development. These results were further supported by Imerovski et al. (2019) [29]. In 2019, Duriez et al. were able to target the first broomrape resistance gene, HaOr7, found on chromosome 7, which encodes a leucine-rich repeat receptor-like kinase [30]. In this study, we examined the resistance of the confectionery hybrid cultivar ‘EMEK3’ (developed by Sha'ar Ha'amakim Seeds, Ltd.), which has high, long-term resistance to sunflower broomrape, with the aim to elucidate—biologically and transcriptomically—the broomrape resistance mechanism, an essential step toward the development of effective sunflower breeding programs.

## 2. Results

### 2.1. Effect of Grafting on the Source of the Resistance

**Figure 1:**
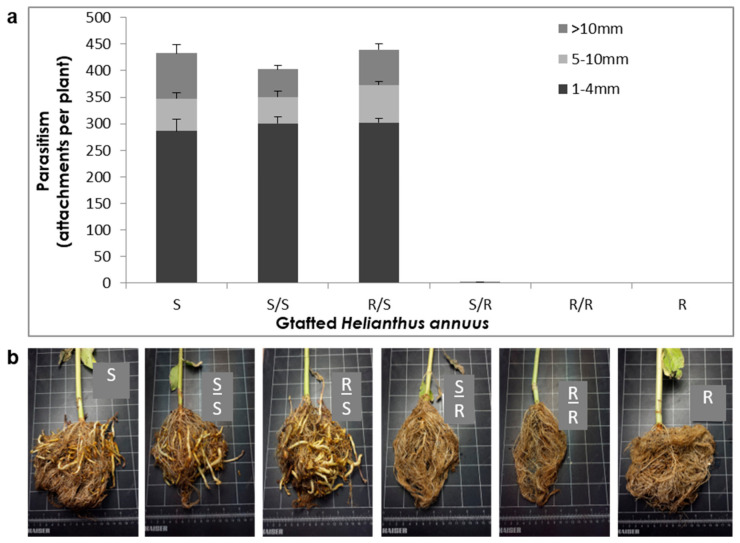
(**a**) Number of *O. cumana* tubercles parasitizing grafted sunflower plants (+SE). S: non-grafted susceptible sunflower; S/S: self-grafted susceptible sunflower; R/S: resistant sunflower shoot grafted onto susceptible sunflower rootstock; S/R: susceptible sunflower shoot grafted onto resistant sunflower rootstock; R/R: self-grafted resistant sunflower; R: non-grafted resistant sunflower. (**b**) Grafted and non-grafted sunflower roots 52 d post-infestation with *O. cumana*.

To test whether biological compounds that are produced in above-ground tissues are involved in the resistance to broomrape, a grafting experiment was conducted. All possible combinations of resistant and susceptible rootstalk/scion grafts were generated, non-grafted plants were used as a control, and the development of the broomrape was monitored. Overall, no parasitism was observed on resistant roots or rootstalks regardless of the type of scion used. All plants with a susceptible root or rootstalk were infested with 420–450 *O. cumana* tubercles and stalks of different sizes, regardless of whether the grafted scions were resistant or susceptible (Figure 1a,b). These results indicate that the aboveground tissues of the sunflower plant do not contribute substantially to its resistance to broomrape.

### 2.2. Sunflower–O. cumana Incompatibility

**Figure 2:**
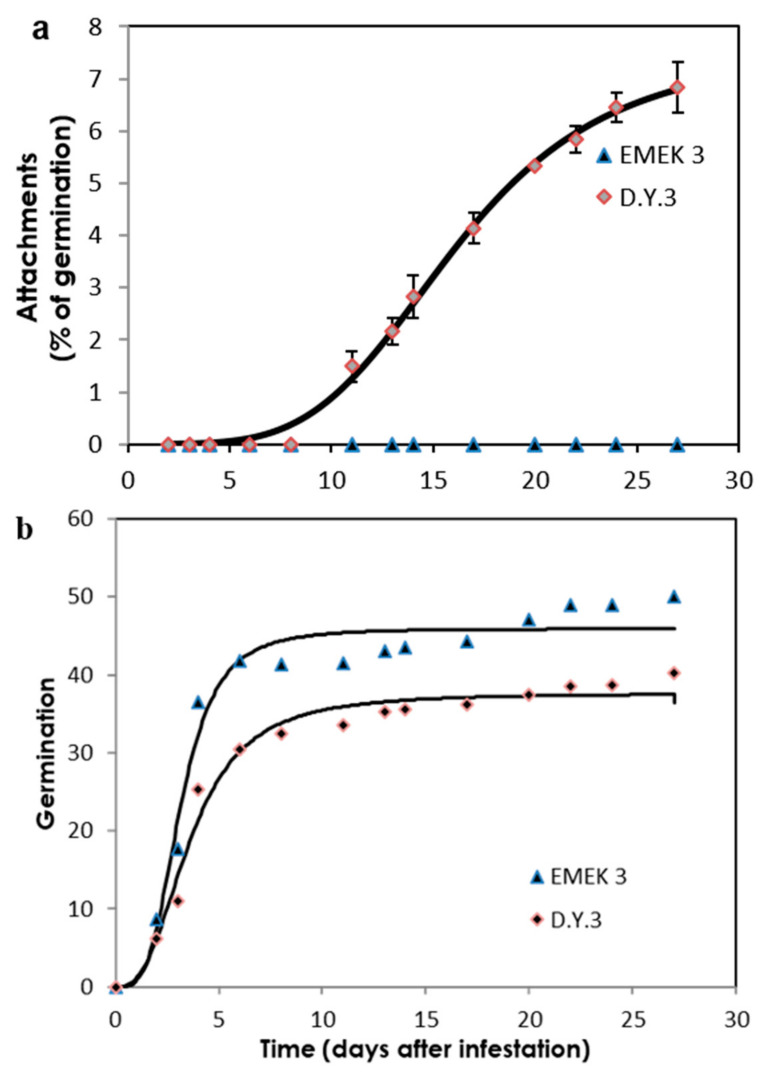
Parasitism dynamics of *O. cumana* on resistant (‘EMEK3’) and susceptible (‘D.Y.3’) sunflowers grown in a polyethylene bag system. Attachment (% of germinated seeds) (**a**) and germination (**b**) of *O. cumana* in the presence of resistant and susceptible sunflower cultivars.

**Figure 3:**
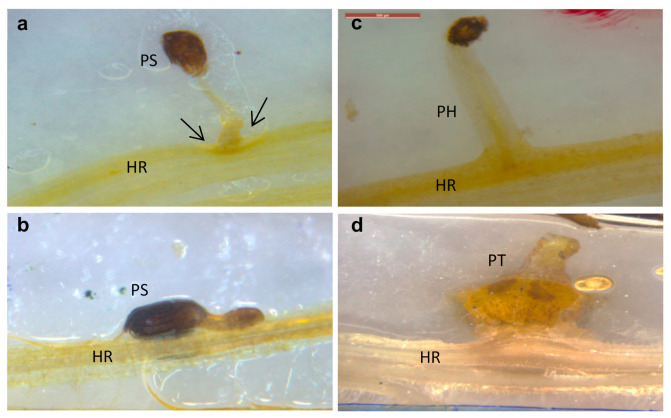
Resistant (‘EMEK3’) (**a**,**b**) and susceptible (‘D.Y.3’) (**c**,**d**) sunflower roots infested with *O. cumana*, 10 (**a**,**c**) and 21 (**b**,**d**) d post infestation. PH: parasite haustorium; HR: host root; PS: parasite seedling; PT: parasite tubercle.

**Figure 4:**
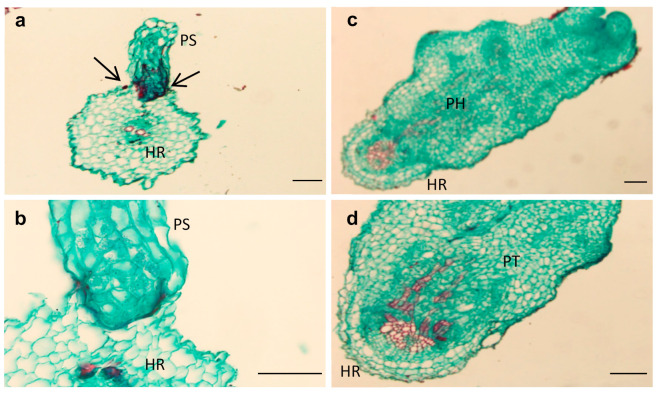
Cross-sections of compatible and incompatible interactions of *O. cumana* with resistant (‘EMEK3’) (**a**,**b**) and susceptible (‘D.Y.3’) (**c**,**d**) sunflower roots five d post-infestation. PH: parasite haustorium; HR: host root; PS: parasite seedling; PT: parasite tubercle. Scale bar = 100 µm.

Two key parasitism stages were monitored periodically: germination and attachments (Figure 2a,b). The germination rate of *O. cumana* seeds was higher (50%) in the presence of the resistant cultivar roots than in the presence of the susceptible cultivar roots (39%) (Figure 2b). The first *O. cumana* attachment was observed 11 d after infestation in the susceptible cultivar, while no attachments were observed in the resistant cultivar (Figure 2a). The observation of *O. cumana* seedlings growing together with sunflower plantlets in clear polyethylene bags (PEB) revealed that the development of the *O. cumana* seedlings was arrested after attaching and attempting to invade the roots of the resistant cultivar, thereby preventing parasite establishment. The disruption of the parasite penetration into the host roots and the subsequent deterioration of the parasite seedlings was accompanied by a darkening of host and parasite tissues at the penetration point (Figure 3a,b). In contrast, establishment and development of healthy tubercles was observed in the roots of the susceptible cultivar (Figure 3c,d). The time at which the resistance response was induced most strongly after the attachment of the parasite seedling to the host roots was determined by observing the host–parasite system growing in the PEB system for 21 d. A large increase in necrotic *O. cumana* seedlings in the presence of the resistant cultivar roots was observed five d after infestation; at this time point, the necrosis of *O. cumana* seedlings that had attached to the resistant cultivar roots grew from 0 to 44% (percentage of germinated seeds), while on the susceptible cultivar roots, only 9% appeared necrotic (Figure S1). Histological examination of ‘EMEK3’ (resistant) and ‘D.Y.3’ (susceptible) roots along with the attached parts of *O. cumana* seedlings, which were sampled five d post infection, showed that the intruding *O. cumana* cells were blocked at the cortex of the resistant cultivar roots and could not reach the endodermis (Figure 4a,b). The root endodermal cells of the resistant cultivar and the attached *O. cumana* seedling-intruding cells were stained with safranin, indicating lignification of cell walls, which presumably prevented the connection of the parasite to the host vascular system and hence, the development of the parasite, whereas in the susceptible cultivar, the formation of the haustorium and compatible connection to the vascular system was observed (Figure 4c,d).

### 2.3. Identification of Candidate Resistance Genes, Using RNA-Sequencing

Comparative RNA-Seq of *O. cumana*-infested and -non-infested sunflower roots of the resistant (E) cultivar, the R bulk, and the S bulk were used to identify differentially expressed genes (DEGs) associated with sunflower resistance. A total of 7.4–9.8 × 10^6^ reads were produced with an average of 8,648,866.3 reads per library.

**Figure 5:**
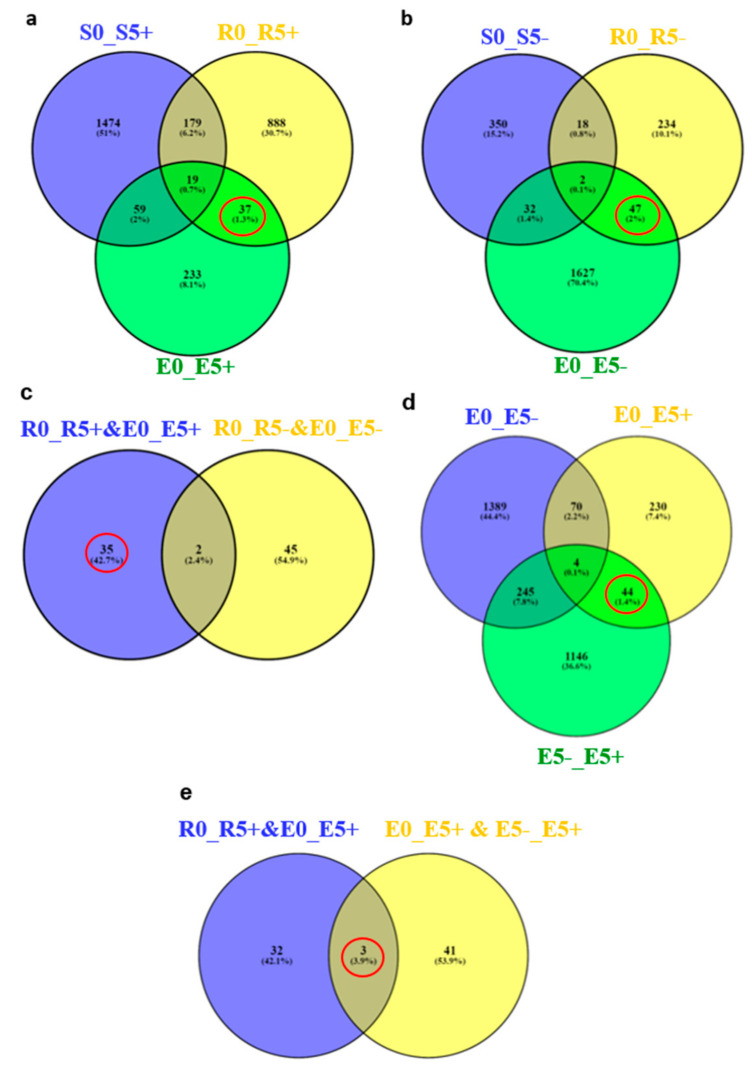
Venn diagram of the DEGs between the R bulk (R), S bulk (S), and EMEK3 (E) pre-infestation (0), five d post-infestation with *O. cumana* (5+) (**a**) and five d post-infestation without *O. cumana* (5−) (**b**). (**c**) Venn diagram of the communal DEGs of (**a**,**b**). (**d**) The DEG in EMEK3 pre- and five d post-infestation with or without *O. cumana*. (**e**) Venn diagram of the communal DEGs of (**c**,**d**).

**Figure 6:**
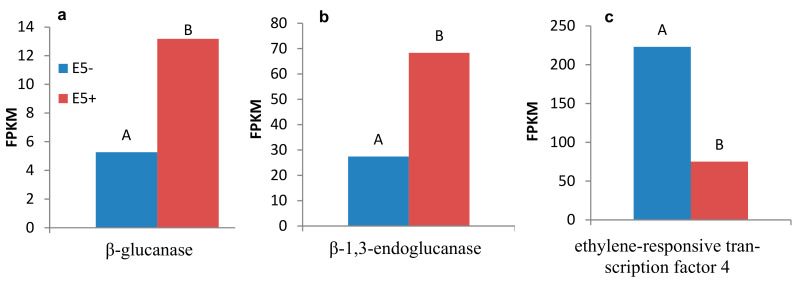
Expression levels (fragments per kilobase million) of the genes encoding β-glucanase (**a**), β-1,3-endoglucanase (**b**), and ethylene-responsive transcription factor 4 (**c**) in EMEK3 roots five d post-infestation with *O. cumana* (E5+) and in non-infested (E5−) roots. Different letters indicate significant difference between groups.

**Figure 7:**
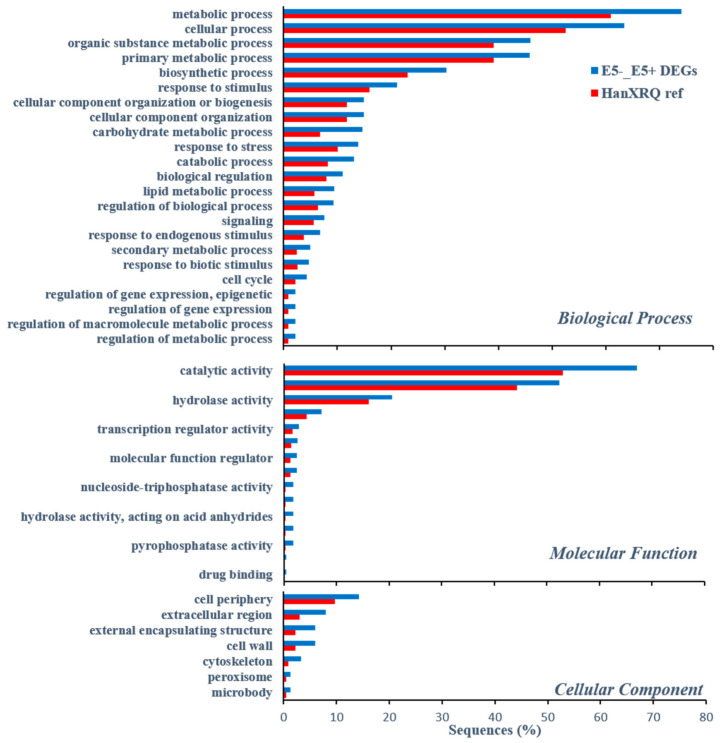
Distribution of enriched GO terms for differentially over-expressed genes (Fisher’s Exact Test) for DEGs of the resistant cultivar EMEK3 [i.e., genes that were differentially expressed between roots of EMEK3 five d post infestation with *O. cumana* (E5+) and non-infested roots (E5-)] compared with GO terms of whole reference-predicted gene annotation (HanXRQ). The Y-axis represents significant enrichment of GO terms and the X-axis shows the relative frequency of the term.

Out of 1123 and 348 genes that were differentially expressed pre-infestation and five d post-infestation in the R bulk and ‘EMEK3’, respectively, 37 genes were found to be communal and not differentially expressed in the S bulk (Figure 5a). To exclude genes that were not related to broomrape infestation, we cross-compared the DEGs of non-infested samples collected on the infestation day and at five d post-infestation: 47 genes were found to be communal to R bulk and ‘EMEK3’ (Figure 5b). These 47 genes were then cross-compared with the 37 genes previously mentioned. Two genes were found to be communal and were therefore discarded (Figure 5c). Hence, 35 genes were classified as related to the resistance response (Figure 5c). Thereafter, we cross-compared the genes that were differentially expressed in ‘EMEK3’ among all treatments: because there was a five-day difference between sampling dates, we assumed that some of the DEGs were not related to the resistance response (i.e., regulatory genes). Therefore, we focused on the 44 genes that were differentially expressed pre-infestation and at the time corresponding to five d post-infestation with and without *O. cumana* (Figure 5d). Finally, these 44 DEGs were cross-compared with the 35 DEGs communal to R bulk and ‘EMEK3’ during the resistance response; 3 genes were found to be mutual (Figure 5e). These three genes were annotated to the sunflower genome and were identified as β-glucanase, β-1,3-endoglucanase, and ethylene-responsive transcription factor 4 (ERF4). The expression levels of the genes encoding β-1,3-endoglucanase and β-glucanase were 2.49 and 2.5 times higher in ‘EMEK3’ roots, respectively (Figure 6a,b). The expression level of the gene encoding ERF4 was 2.97 times lower in ‘EMEK3’ roots five d after the infestation with *O. cumana* (Figure 6c). Gene ontology (GO) enrichment analysis, performed on 1439 significant DEGs in the resistant cultivar, showed that 224 overexpressed genes were significantly enriched (false discovery rate (FDR) < 0.05). The most enriched term in the *Biological Process* class was “metabolic process” (74%). For the *Molecular Function* and *Cellular Component* classes, these terms were “catalytic activity” (67%) and “cell periphery” (14%), respectively (Figure 7).

## 3. Discussion

A variety of strategies comprising the host defense response at the early stages of the parasite life cycle have been described in a number of studies, namely, the lignification and subarization of host cell walls [10]; the accumulation of callose, peroxidases, and H_2_O_2_ in the cortex and protein cross-linking in the cell walls [11,12]; phenylalanine ammonia lyase (PAL) activity and high concentrations of phenolic compounds in the host roots [31,32,33]; and degeneration of tubercles after establishment [14]. Determining the phenological stage at which the incompatibility occurs is crucial for understanding the resistance mechanism and the molecular basis that governs it. To this end, we set up an observation system based on transparent PEBs that enabled us to follow the sunflower–broomrape interaction continuously and thereby to overcome the difficulty of detecting the exact time at which response was maximal. Our observations revealed that the resistance response began in the early stages of the parasite life cycle, after germination and attachment to the roots, and while the parasite was attempting to penetrate into the host roots (Figure 2, Figure 3 and Figure 4). Blocking of the penetration attempt was accompanied by the necrosis of parasite and host tissues in the penetration area, suggesting a pre-haustorial mechanism of resistance [12]. Our PEB system showed a markedly high necrosis rate of the attached broomrape seedlings in the roots of the resistant cultivar at five d post-infestation (Figure S1). This high death rate was attributed to the prevention of penetration into the host roots and hence, prevention of the establishment in the vascular system that is vital for the parasite seedlings. We thus confirmed by histological methodologies that the parasite intrusion was blocked in the host cortex before the parasite could reach the host endodermis. The endodermal cells in the vicinity of the intrusive broomrape cells in the penetration area were colored with safranin, indicating the involvement of lignin in the host response (Figure 4). Suberization, lignification, and cell wall thickening have previously been ascribed to the sunflower defense response to *O. cumana* [10,34,35]. We excluded the possibility that the host shoot was involved in the resistance response by grafting susceptible sunflower scions onto resistant rootstocks and vice versa. The resistant cultivar rootstocks conferred resistance on the susceptible scions, but the susceptible rootstocks were parasitized with *O. cumana* regardless of whether the grafted scions were resistant or susceptible (Figure 1). Similar results have been obtained for the resistance of the tomato (*Solanum lycopersicum*) to several broomrape species, namely, *P. aegyptiaca*, *P. ramosa*, *O. cernua*, and *O. crenata* [36], implying that the resistance response is expressed exclusively in the roots. A comparative transcriptome analysis of infested and non-infested resistant and susceptible sunflower roots detected 1439 significant DEGs in the roots of the resistant cultivar post-infestation. GO enrichment analyses of these DEGs were performed to infer the biological processes and the functions of the genes associated with the resistance response, with the ontology analysis revealing a number of overexpressed GO terms (Figure 7). Importantly, terms associated with the cell periphery (14%), the extracellular region (7.9%), the external encapsulating structure (5.9%), and the cell wall (5.9%) were significantly enriched in the *Cellular Component* category, indicating high activity in these regions. The *Biological Process* category included response to stimulus (21%), cellular component organization (15%), and response to stress (13.8%) (Figure 7). Finally, a series of Venn diagrams [37] facilitating cross-comparisons of DEGs in the R bulk, the S bulk, and the resistant cultivar ‘EMEK3’ before and after infestation with *O. cumana* identified three genes that were differentially expressed between infested and non-infested sunflower roots of both ‘EMEK3’ and the R bulk (Figure 5). As a consequence of the infestation, two of these genes, β-1,3-endoglucanase and β-glucanase, were upregulated, and the third gene, ERF4, was downregulated. These findings indicate activation of the plant's innate immune system, in which the recognition of PAMPs activates a hypersensitive response and the accumulation of pathogenesis-related (PR) proteins [38,39] such as β-glucanases, which are PR proteins belonging to the PR-2 family. This family of proteins is believed to play an important role in plant defense responses to pathogen infection [40,41,42]. Indeed, it has been shown that β-glucanases, which are able to degrade cell wall β-glucan, are involved in resistance to *O. crenata* in peas (*Pisum sativum*) [11,43] and in sunflower resistance to *O. cumana* [33]. Downregulation of the ERF4 gene post-infection should be viewed in the context of the role of the endogenous hormone, ethylene, in regulating defense responses in plants, including the regulation of gene expression during adaptive responses to abiotic and biotic stresses [44]. The ERF transcription factors, which are unique to plants, have a binding domain that can bind to the GCC box, an element found in the promoters of many defense, stress-responsive, and PR genes [45,46]. Just as there is a range of stresses, there are a large a number of ERFs, with many of the ERFs being transcription activators. Indeed, AtERF1, AtERF2, and AtERF5 act as transcriptional activators, although AtERF3 and AtERF4 act as transcriptional repressors for GCC box-dependent transcription in Arabidopsis leaves [47]. In that context, McGrath et al. (2005) [48] demonstrated that the Arabidopsis *erf4-1* mutant was resistant to *Fusarium oxysporum*, while transgenic lines overexpressing AtERF4 were susceptible, and therefore concluded that AtERF4 negatively regulates resistance to *F. oxysporum*. The downregulation of the ERF4 gene in the roots of the resistant sunflower post-*O. cumana* infestation suggests that, as in Arabidopsis, the sunflower response to biotic stress is negatively regulated by ERF4. Furthermore, the recent study of Liu et al. (2020) [49], using bulked segregant RNA-Seq (BSR-Seq), identified ERF as a candidate gene for *O. cumana* resistance in sunflowers. Taken together, the results obtained for the biological characterization combined with those for the genetic characterization provide a comprehensive view of the relations between the resistant cultivar ‘EMEK3’ and *O. cumana*. This broad view allowed us to propose the following resistance mechanism model: After ‘EMEK3’ induces *O. cumana* seed germination, the seedlings’ attachment to the sunflower roots is perceived by PAMPs. These molecules set off a PTI response that downregulates ERF and abrogates the suppression of PR genes (including β-glucanase). β-glucanase then breaks down the parasite cell walls which, in turn, release effectors that trigger the second level of the plant immune response, namely, effector-triggered immunity (ETI). As a result, a physical barrier is created by the accumulation of lignin and other phenolic compounds in the penetration area, and the *O. cumana* seedlings fail to establish a connection with the host vascular system, leading to parasite necrosis.

## 4. Conclusions

Sunflower pre-haustorial resistance to sunflower broomrape involves the expression of β-1,3-endoglucanase, β-glucanase, and ethylene-responsive transcription factor 4 (ERF4) genes. The resistance mechanism includes the identification of the broomrape and the formation of a physical barrier that prevents the penetration of the broomrape into the sunflower roots.

## 5. Materials and Methods

### 5.1. Plant Materials and Growth Conditions

The cultivars ‘EMEK3’ (resistant) and ‘D.Y.3’ (susceptible) as well as 12 sunflower breeding accessions that are being used in breeding programs for the introgression of different traits for the development of Israeli sunflower cultivars were kindly provided by Sha’ar Ha’amakim Seeds, Ltd. (Sha’ar Ha’amakim, Israel). *O. cumana* inflorescences were collected from an infested sunflower field in northern Israel in 2012. The seeds were separated from the capsules, using 300-mesh sieves, and stored in the dark at 4 °C prior to use. The germination rate of these *O. cumana* seeds at 25 °C was 85%.

### 5.2. Preconditioning of O. cumana Seeds

Preconditioning was performed under sterile conditions. The seeds were surface sterilized for 2.5 min in ethanol (70%) followed by 10 min in sodium hypochlorite (1%) and then rinsed 5 times with sterile distilled water and dried for 2 h in a laminar airflow cabinet. The dried seeds were spread on 5.5 cm diameter glass fiber filter paper discs (Whatman #3, Whatman International, Ltd., Maidstone, England) that had been wetted with 600 µL of sterile distilled water. The discs were placed in sterile, 5.5 cm diameter petri dishes. The petri dishes were sealed with Parafilm and incubated at 25 °C for 7 d in the dark. Thereafter, 220 µL (10^−5^ M) of GR24 (a commonly used broomrape synthetic germination stimulant [6]) were added to the discs, and the petri dishes were resealed and kept in the dark for another 24 h.

### 5.3. Cultivation in Polyethylene Bag

The polyethylene bag (PEB) system of Parker and Dixon (1983) [50], with the slight modification of Eizenberg et al. (2003) [51] to tailor it to sunflower cultivation, was used for observing the sunflower–broomrape interaction, as follows: Sunflower seedlings at the cotyledon stage were placed on 25 × 10 cm glass microfiber filter papers (Whatman GF/A), which were then inserted into clear PEBs (35 × 10 cm) and allowed to grow in a growth chamber under controlled conditions (25 °C; 18 h light; 150–200 µE m^−1^ s^−1^) for 10 d. Approximately 5 µg of preconditioned *O. cumana* seeds were then carefully placed alongside the sunflower roots on the GF/A filter papers. Sterilized, half-strength Hoagland nutrient solution [52] (5 mL) was supplied every day. Observations were carried out every 2–3 d with an electronic binocular microscope (Leica M80) to monitor seed germination, attachment, and establishment or necrosis of the *O. cumana* seedlings.

### 5.4. Histological Analysis

Plant material for histological analysis was taken from the PEB system. ‘EMEK3’ and ‘D.Y.3’ roots, along with the attached parts of *O. cumana* seedlings, were sampled five d post-infection. In parallel, non-infected sunflower roots (control) were sampled. The sampled roots (with and without *O. cumana*) were fixed in FAA (5% formalin:5% acetic acid:90% alcohol (70%), *v/v*) for 24 h. Fixed samples were then dehydrated in an ethanol series (50, 70, 90, 95, 100%; 1–2 h each). After dehydration, the samples were infiltrated with a series of Histo-Clear:ethanol (1:3, 1:1, 3:1 ratio; 1 h each), cleared with Histo-Clear (xylene substitute), and embedded in paraffin. The samples were then cut into 13µm sections with a rotary microtome (Leica RM2245, Leica Biosystems, Nussloch, Germany) and stained with safranin/fast green [53].

### 5.5. Grafting Experiments

To assess the involvement of the shoot in the resistance mechanism, grafting experiments were conducted as follows: ‘EMEK3’ and ‘D.Y.3’ seeds were sown in 2-liter pots, and 14 d post-emergence, the stems of plants with two true leaves were cut above the cotyledons at a 45° angle. ‘EMEK3’ shoots were grafted onto ‘D.Y.3’ rootstock and vice versa. The grafted sunflowers were kept in a closed chamber with 100% humidity at 25 °C for 3 d. The plants were then transferred to a humid chamber (in which water was sprayed every 3 h for 10 s) for 7 d. Thereafter, the plants were covered with polyethylene and were gradually exposed to the room atmosphere by removing portions of the polyethylene cover every 1–2 d along 7 d. Once plants were acclimated, they were planted in 2-liter pots and infested with 15 ppm of *O. cumana* seeds. Self-grafted and non-grafted plants were used as a control in the experiment.

### 5.6. Statistical Analysis

The experiments were carried out in 5 replications in a fully randomized design. The analysis of variance (ANOVA) was performed, and means were compared using the Student’s t test (*p* < 0.05) in JMP PRO 12 software (v5.1; SAS Institute, Inc., Cary, NC, USA).

### 5.7. Bulk Construction and RNA Extraction

Twelve sunflower breeding accessions that had been used as a genetic source for ‘EMEK3’ breeding were quantified for *O. cumana* resistance under conditions of artificial infestation in pots held in a greenhouse (25–30 °C). Five accessions showed complete resistance with no attachments on the roots, and seven accessions exhibited susceptibility at all *O. cumana* parasitism stages (Figure S2). Therefore, in addition to the resistant cultivar, five resistant accessions and five susceptible accessions were selected to construct a resistant (R) bulk and a susceptible (S) bulk for RNA sequencing. Roots of PEB-cultured sunflowers of five resistant accessions, five susceptible accessions, and the resistant cultivar were collected on the day of infestation and at five d post-infestation with *O. cumana* for both infected and control plants. Whole roots were ground in liquid nitrogen, and equal amounts of root tissue from each accession of the resistant and the susceptible accessions were taken as R and S bulks. Total RNA was isolated from 27 samples (‘EMEK3’, R bulk, and S bulk × 3 treatments/sampling time × 3 replicates), using a Spectrum^TM^ Plant Total RNA Kit (Sigma-Aldrich, St. Louis, MO, USA) according to the manufacturer’s protocol. RNA quality and integrity were evaluated by Agilent TapeStation 2200 (Agilent Technologies, Santa Clara, CA, USA).

### 5.8. RNA Sequencing and Mapping

Libraries were prepared using the Genomics in-house protocol for mRNA-seq. Briefly, the polyA fraction (mRNA) was purified from 500 ng of total RNA, followed by fragmentation and the generation of double-stranded cDNA. Next, end repair, a base addition, adapter ligation, and PCR amplification steps were performed. Libraries were evaluated by Qubit (Thermo Fisher Scientific) and TapeStation (Agilent). Sequencing libraries were constructed with barcodes to allow multiplexing of 27 samples in 2 lanes. Approximately 16–20 million single-end 60-bp reads were sequenced per sample on an Illumina HiSeq 2500 V4 instrument. The quality of the raw reads was evaluated using FastQC v.0.11.03 [54], followed by trimming and removal of low-quality reads, using Trimmomatic v.036 [55]. Cleaned reads from each of the 27 libraries were then aligned to the *H. annuus* XRQ v1.0 reference genome [56], using STAR v.2.5.2b [57], and the level of expression of each gene in each library was estimated using RSEM v.1.2.31 [58]. Expression levels were normalized using the number of reads per kilobase per million reads mapped (RPKM) for each transcript.

### 5.9. RNA-Seq Data Analysis

The RSEM output files were analyzed using R package DESeq2 [59] for differential expression analysis. A pairwise comparisons test was performed between ‘EMEK3’, R bulk, and S bulk. DEGs were considered as significant at FDR < 0.05 [60]. GO terms were obtained from the heliagene database for XRQ (https://www.heliagene.org/HanXRQ-SUNRISE/, accessed on 27 December 2016), and GO terms enrichment analysis was performed for significant DEGs compared to all other GO terms using the Blast2GO (v5.2.5) analysis tools [61]. Significantly over-represented GO terms were identified using Fisher’s exact test at a significance level of FDR < 0.05. GO slim (Blast2GO tool) was performed to reduce the complexity of GO terms for functional analysis of annotated *H. annuus* genes.

## Data Availability

The data from RNA-seq has been submitted into the NCBI SRA database, and the BioProject accession number is PRJNA706194 (https://www.ncbi.nlm.nih.gov/sra/PRJNA706194, accessed on 27 May 2021).

## Supplementary Materials

The following are available online at www.mdpi.com/article/10.3390/plants10091810/s1, Figure S1: Sunflower-*O. cumana* incompatibility, Figure S2: Screening of sunflower breeding ac-cessions for resistance to *O. cumana*.
