# Supplementary figures and images for: Biological and Transcriptomic Characterization of Pre-Haustorial Resistance to Sunflower Broomrape (Orobanche cumana W.) in Sunflowers (Helianthus annuus)
Source: Plants (Basel). 2021 Aug 30;10(9):1810. doi: 10.3390/plants10091810 (PMC8465872; doi:10.3390/plants10091810)

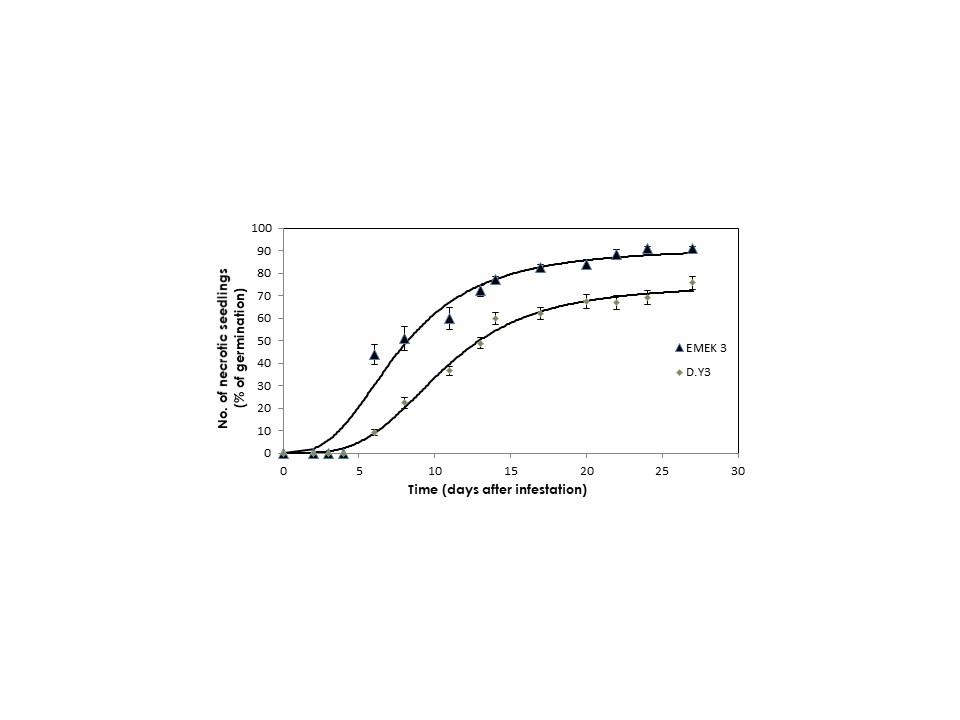

Supplement: Supplementary file 1 [file plants-10-01810-s001.zip › Supplementary1.JPG]

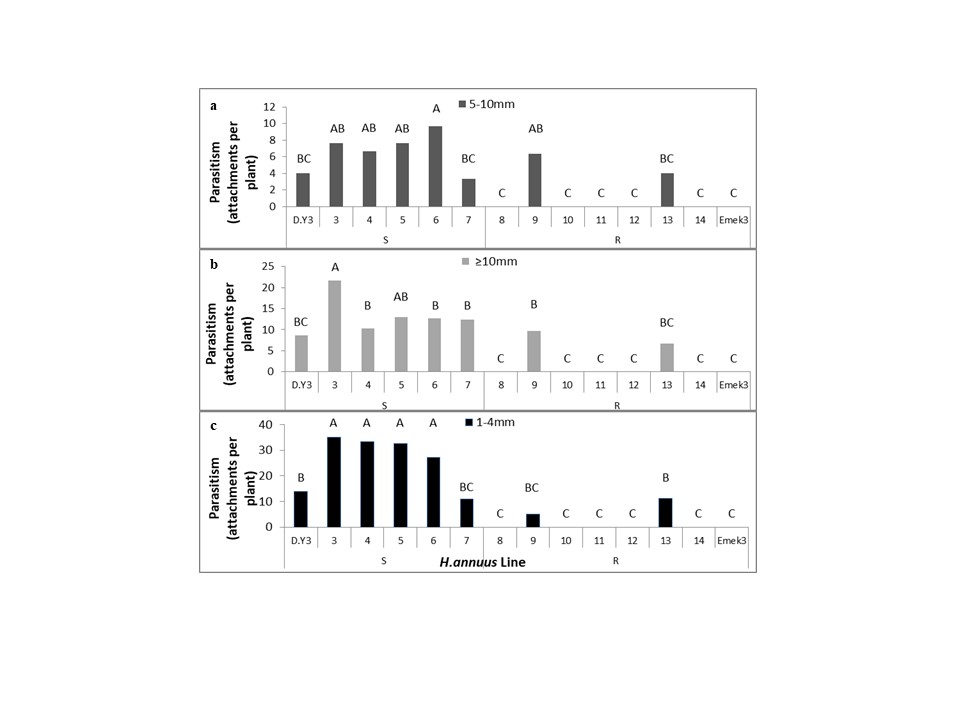

Supplement: Supplementary file 1 [file plants-10-01810-s001.zip › Supplementary2.JPG]
